# Corrigendum: Dynamic nuclear magnetic resonance field sensing with part-per-trillion resolution

**DOI:** 10.1038/ncomms14746

**Published:** 2017-02-27

**Authors:** Simon Gross, Christoph Barmet, Benjamin E. Dietrich, David O. Brunner, Thomas Schmid, Klaas P. Pruessmann

Nature Communications 7 Article number:13702 ; DOI: 10.1038/ncomms13702 (2016); Published 12 02 2016; Updated 02 27 2017

The financial support for this Article was not fully acknowledged. The acknowledgements should have included the following: The authors acknowledge Nano-Tera.ch for financial support.

