# Supplementary material for: Dynamic nuclear magnetic resonance field sensing with part-per-trillion resolution
Source: Nat Commun. 2016 Dec 2;7:13702. doi: 10.1038/ncomms13702 (PMC5146285; doi:10.1038/ncomms13702)
Supplement: Supplementary Information — Supplementary Figures 1-4 [file ncomms13702-s1.pdf]

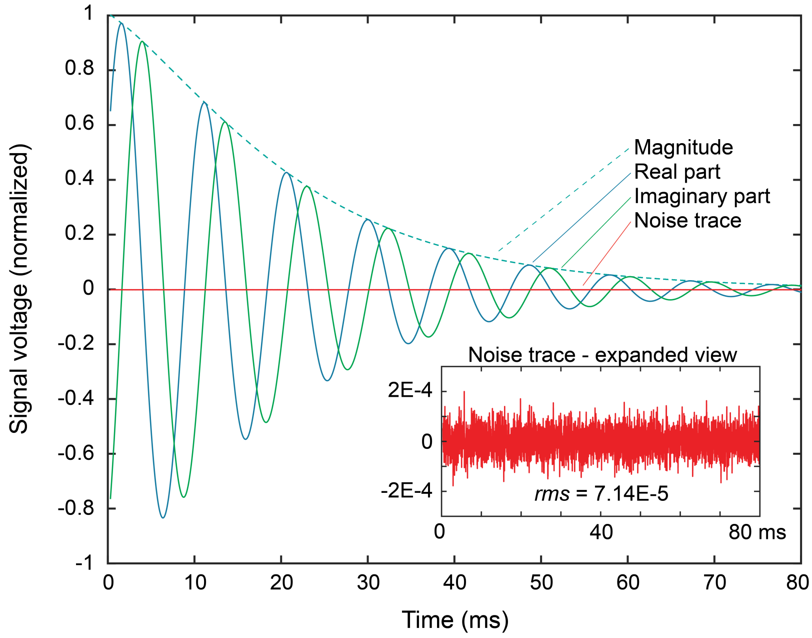

Supplementary Figure 1 | Free induction decay signal and noise trace of the utilized field sensors. The figure shows the acquired signals of the sensors described in Figs. 1 and 2, with and without prior signal excitation. They were acquired using the same spectrometer settings. The normalized signals are proportional to the measured voltages at the analogue-to-digital converter. Such data can be used to determine the signal-to-noise ratio (SNR) at the used bandwidth (50 kHz) which amounts to 1/7.14E-5 = 14,006 for the shown dataset. It corresponds to a bandwidth-compensated SNR of $\xi = 3.13\text{x}10^6\ \text{Hz}^{1/2}$.

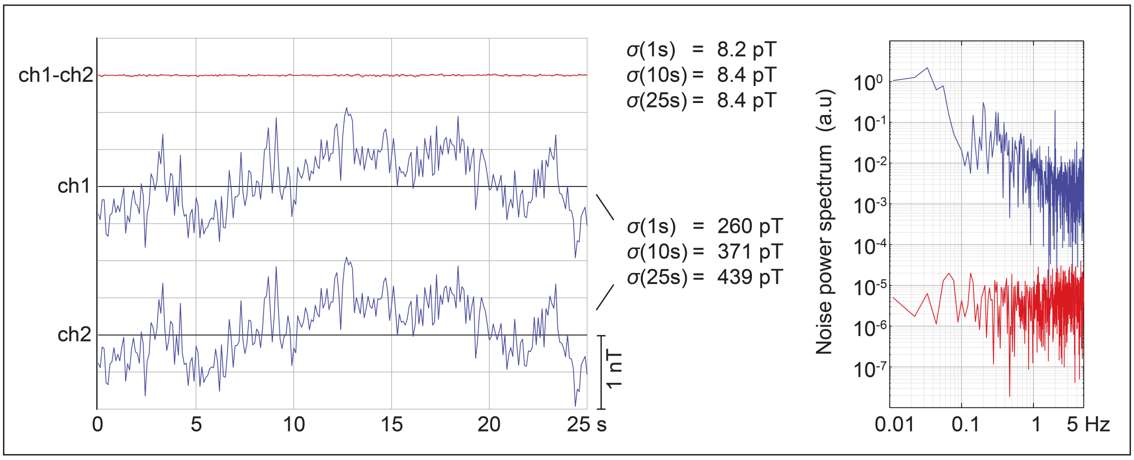

Supplementary Figure 2 | Spectrometer stability measurement using a single sensor. To determine the additional field noise introduced by the spectrometer, we acquired the signal of a single, $CuSO_4$-doped H_2O field sensor, fed through two receive channels. Signal splitting was achieved with a 90° hybrid coupler, inserted before the first amplification stage. Whereas the field traces of the individual channels (blue) reflect the temporal evolution of the magnet and shows the expected non-thermal noise structure, their difference (red) is solely due to additional white spectrometer noise. It amounts to $8.4/\sqrt{2} = 6$ pT per channel.

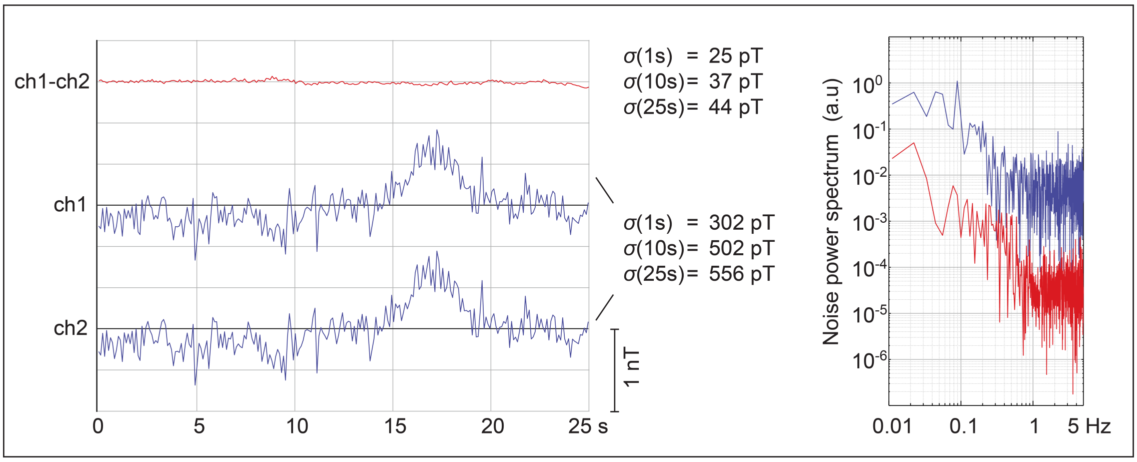

Supplementary Figure 3 | Field stability measurement using two closely spaced sensors. The two field traces (blue) reflect the temporal instability of the magnet with its expected non-thermal noise statistics. The difference trace (red) is substantially more stable. However, it still exhibits structured noise behaviour indicating the presence of non-thermal noise, as caused by spatially non-uniform field fluctuations.

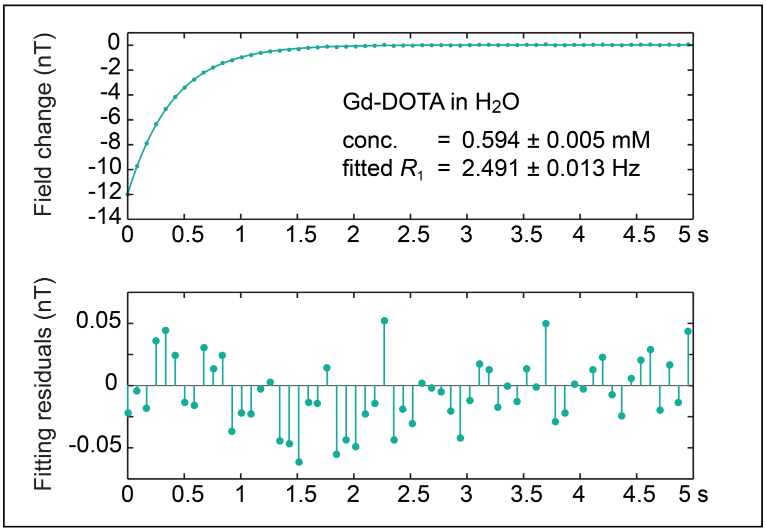

Supplementary Figure 4 | Fitting residuals of direct longitudinal relaxometry of a gadoteric acid solution. Mono-exponential fitting using non-linear least squares yielded the relaxation rate of 2.491±0.013 Hz for a concentration of 0.594±0.005 mM gadoteric acid (Gd-DOTA) in water. The coefficient of determination is R^2 = 0.9998 with the residuals amounting to a root-mean-square error of 26 pT.
